# Cross-Cultural Agreement in Facial Attractiveness Preferences: The Role of Ethnicity and Gender

**DOI:** 10.1371/journal.pone.0099629

**Published:** 2014-07-02

**Authors:** Vinet Coetzee, Jaco M. Greeff, Ian D. Stephen, David I. Perrett

**Affiliations:** 1 Department of Genetics, University of Pretoria, Pretoria, South Africa; 2 Department of Psychology, Macquarie University, Sydney, Australia; 3 School of Psychology and Neuroscience, University of St Andrews, St Andrews, Scotland; Brock University, Canada

## Abstract

Previous work showed high agreement in facial attractiveness preferences within and across cultures. The aims of the current study were twofold. First, we tested cross-cultural agreement in the attractiveness judgements of White Scottish and Black South African students for own- and other-ethnicity faces. Results showed significant agreement between White Scottish and Black South African observers' attractiveness judgements, providing further evidence of strong cross-cultural agreement in facial attractiveness preferences. Second, we tested whether cross-cultural agreement is influenced by the ethnicity and/or the gender of the target group. White Scottish and Black South African observers showed significantly higher agreement for Scottish than for African faces, presumably because both groups are familiar with White European facial features, but the Scottish group are less familiar with Black African facial features. Further work investigating this discordance in cross-cultural attractiveness preferences for African faces show that Black South African observers rely more heavily on colour cues when judging African female faces for attractiveness, while White Scottish observers rely more heavily on shape cues. Results also show higher cross-cultural agreement for female, compared to male faces, albeit not significantly higher. The findings shed new light on the factors that influence cross-cultural agreement in attractiveness preferences.

## Introduction

Facial attractiveness plays a crucial role in a variety of social interactions, from dating [1] to voting behaviour [2]. Historically, different cultures were believed to have different standards of physical attractiveness (e.g. [3]). More recent work, including a meta-analysis of facial attractiveness preferences, found high consistency between people's judgements of facial attractiveness within and across cultures, leading to the conclusion that “raters agree about who is and is not attractive, both within and across cultures” [4]. Most of the studies of adults included in the cross-cultural part of the meta-analysis tested agreement between people of different ethnic origins currently living within a single country. Nevertheless, a few studies tested agreement across different cultural and ethnic groups living in different countries [5]–[9], providing a more stringent test of the universality of attractiveness standards. We will focus only on these latter studies here.

Three studies, Zebrowitz et al. [8], Jones and Hill [6] and Zebrowitz et al. [9], are especially noteworthy because of the quality and size of their image sets. Zebrowitz et al. [8] collected black and white yearbook images of 24 Korean, 20 White American and 24 African American male college students and had all the images rated for facial attractiveness by members of the same three ethnic groups. The Korean raters resided in Korea, while the White and African American raters resided in America. They found high inter-rater reliability in attractiveness judgements across the Korean and American groups (Cronbach α>0.8; [8]). Reliability statistics by themselves do not, however, provide a complete picture of the relationship between the perceptions of different groups of judges [10]. Zebrowitz et al. [8] also tested the correlation between the mean attractiveness judgements of the different ethnic groups, to assess interracial agreement in attractiveness judgements. They found that judges agreed more strongly on what is attractive in own-race faces (calculated by randomly dividing each group of raters in half and correlating the mean ratings of the two subgroups), compared to other-race faces [8].

Jones and Hill [6] collected standardised male and female facial images of White American college students, Brazilian college students and adult Paraguayan Indians. Members from the same three populations, Russian college students and adult Venezuelan Indians rated all the facial images for attractiveness. They found high inter-rater reliability in attractiveness judgements within groups (Cronbach α>0.7), except when Paraguayan and Venezuelan Indians judged Paraguayan Indian male faces for attractiveness. The correlational analyses found that attractiveness judgements within the Western student cluster (White American, Brazilian and Russian students) and the Indian adult cluster (Paraguayan and Venezuelan Indians) were highly correlated, but between clusters the correlation coefficients were much lower and mostly non-significant [6].

Zebrowitz et al. [9] compared facial attractiveness preferences between White American college students and the culturally isolated Tsimane people from the Bolivian rainforest. They collected black and white facial images of American men and colour facial images of Tsimane men. Groups of judges from each population were asked to judge own-and-other ethnicity faces for attractiveness. Both American and Tsimane judges agreed more strongly on what is attractive in American compared to Tsimane faces, although not significantly so in either group [9].

Very few studies have compared facial attractiveness judgements between African nationals and individuals from developed countries. Martin [7] asked Black Nigerians, White Americans and African Americans to judge the facial attractiveness of a small unstandardised set of 10 magazine images of ‘presumably’ black women. Surprisingly, they found higher agreement between Black Nigerians and White Americans than between Black Nigerians and African Americans when judging black female images [7]. Silva et al. [11] found significant cross-cultural agreement in facial attractiveness judgements between rural Senegalese and British judges when judging a small subsample (N = 16) of American faces. To our knowledge, no previous study has tested cross-cultural agreement in attractiveness preferences between African nationals and individuals from a Western country for own- and other-ethnicity faces.

It is clear that different cultures show significant agreement in what is considered attractive, but there is also reason to expect fine scale differences in agreement between cultures. Comparatively few studies have investigated the factors that could influence cross-cultural agreement in attractiveness preferences. For one, cultural differences in the utility of information gleaned from the face ― such as how accurately attractiveness reveals health or fertility ― could influence agreement between cultures [12]. Perceptual experience could also influence cross-cultural agreement in attractiveness preferences. A person's notion of a ‘prototypical’ or ‘average’ face depends on the faces they have been exposed to during their lifetime [13]. Average faces are generally considered more attractive (e.g. [14]) so faces closer to the person's ‘prototype’ face should be considered more attractive. Furthermore, individuals often show preferences for self-resembling [15], [16] and parental traits [17] in their prospective partners ― especially traits associated with the opposite sex parent [18], [19]. These preferences have been attributed to assortative mating (selection of a mate with preference for a particular phenotype), but could also be more generally attributed to perceptual narrowing during childhood. Perceptual narrowing is a decrease in the discrimination ability between objects to which we are not regularly exposed during certain critical times of our development. For example, in one study, three-month-old human infants could discriminate between individual images of humans and monkeys, but by the age of nine months infants could discriminate only between human images [20].

One example of perceptual narrowing in humans is the ‘own race bias’ or ‘cross-race effect’. According to the ‘own-race bias’, people are better at recognising and discriminating between faces from their own ethnicity compared to faces from other ethnicities [21]–[23]. This ‘own-race bias’ develops very early in life [24], presumably due to increased exposure to own-race faces during development. Exposure to other-ethnicity faces during development [25] and later in life can reverse the own-race bias to some extent. Adults that were adopted from Korean families between the ages of three and nine years, and raised by French families, were significantly better at recognising Caucasian faces than Asian (Japanese) faces [26]. It follows that people who have more interracial contact are better at discriminating between and recognising faces from other ethnic groups (for meta-analytic review see [23]). One might argue that interracial contact also increases people's perceptual expertise in other areas, such as the perception of attractiveness. Indeed, facial recognition and likeability/attractiveness judgement tasks stimulate similar brain regions [27] and facial attractiveness influences facial recognition memory; highly attractive and unattractive faces are recognised significantly better than moderately attractive faces 35 days after exposure [28].

## Experiment 1

The first aim of this study is to test cross-cultural agreement in the attractiveness judgements of White Scottish and Black South African students for own- and other-ethnicity faces using a large set of standardised full-colour images. The second aim is to test whether two factors, the ethnicity and gender of the target group, influence cross-cultural agreement in attractiveness preferences. Black South Africans are regularly exposed to White European facial features, since 9.2% of the South African population are of European descent [29] and Western media influences are highly pervasive in South Africa. Black students at the University of Pretoria are particularly exposed to White European facial features, since 53% of the contact students at this university are of European descent (Unpublished data, University of Pretoria management information, November 2012). In contrast, only 0.12% of the Scottish population is classified as African or other Black [30] and only about 2% of the contact students at the University of St Andrews are African. This discrepancy in perceptual exposure to other race faces is also expected to be evident during early development, when perceptual narrowing takes place [24]. It follows that Black South African and White Scottish observers should show higher cross-cultural agreement for White faces, compared to Black faces, because both groups of observers have developed perceptual expertise for White European faces. We also predict higher cross-cultural agreement for female than for male faces. Men value physical attractiveness in a partner more than women do, irrespective of their cultural background [31]. Women's attractiveness judgements of male faces might therefore also be influenced by other factors, such as apparent socio-economic status, which might weaken cross-cultural agreement for male faces. Furthermore, men show a robust preference for femininity in female faces, while women's preferences for male facial masculinity are variable (for review see [32]). For example, previous work found that pathogen load (or more generally ill health; [33]–[35] and/or income inequality [36]) positively influence women's preference for masculinity in male facial images. One might therefore also expect higher cross-cultural agreement in the attractiveness judgements of female, compared to male, faces.

### Methods

#### Ethics statement

**Figure 1:**
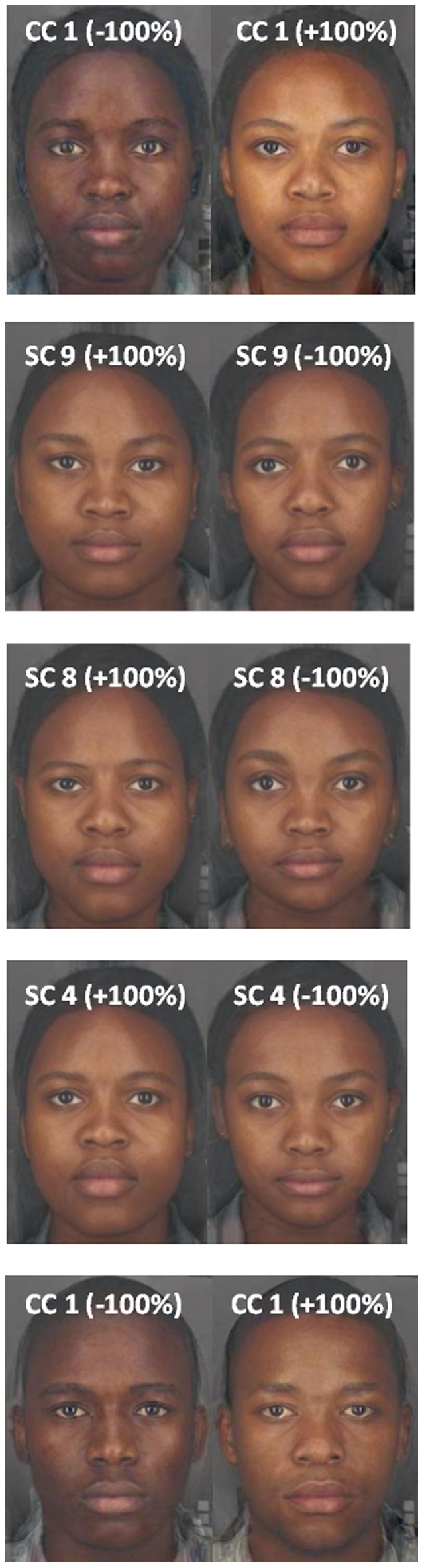
Visualisation of the shape and colour components. Composite faces were transformed to illustrate exaggerated positive (+100%) and negative (−100%) dimensions of each significant and marginally significant shape and colour component. Images were arranged so that images on the right hand side always indicate the more attractive dimension. CC = colour component; SC = shape component.

This study was approved in writing by ethics committees at the University of Pretoria (EC090304-020, EC090803-045) and the University of St Andrews (PS3137, PS5199, PS5740). All participants gave written informed consent prior to taking part in the study and were debriefed after participation. The individuals whose images were used to produce the composite images in Figure 1 have given written informed consent to have their images used in publication.

#### Photography

We collected full-colour facial images of 96 Black South African participants (47 male; Mean Age±SD = 19.83±2.14) from the University of Pretoria (hereafter African images) and 83 White participants (40 male; Mean Age±SD = 21.13±1.68) from the University of St Andrews (hereafter Scottish images). Both image sets were used in previous studies. Coetzee et al. [37] and Stephen et al. [38] provide a full description of the standardised image capture, delineation and alignment methods for the African images, while Coetzee et al. [39] provides a description for the Scottish images. Participants reported their sex and age.

#### Experimentation

We recruited a total of 226 African and Scottish participants to rate the facial images for attractiveness. African observers were recruited from the University of Pretoria and Scottish observers from the University of St Andrews. The African observers were divided into four groups: a group judging African female faces; a group judging African male faces; a group judging Scottish female faces; and a group judging African male faces (Table 1). Scottish observers were divided into four similar groups (Table 1). Observers reported their age and sex, and were asked to indicate whether they knew image participants if they were from the same university. Images were presented in a randomised order on colour-calibrated monitors and rated for attractiveness on seven point Likert scales. All observers used a point rating scale, with higher values indicating higher attractiveness. Once the attractiveness judgement was made the next image would be displayed.

**Table 1:** Descriptive statistics for different observer groups.

	African observers		Scottish observers	
	N	Age	N	Age
*African images*				
Female	30 (14 male)	20.28±1.78	32 (13 male)	20.56±1.72
Male	29 (14 male)	20.75±2.63	30 (10 male)	20.60±1.63
*Scottish images*				
Female	29 (11 male)	20.90±1.76	26 (12 male)	22.81±2.02
Male	27 (10 male)	20.47±2.06	23 (10 male)	21.24±2.21

Age indicated as Mean±SD. N refers to the number of observers.

#### Statistical analyses

We excluded three observers because they rated all images in the image set equally attractive (1 African male rating African male faces and 2 African males rating Scottish male faces). Attractiveness ratings were averaged across image participants for each of the two rater groups. All variables were examined for accuracy of data entry, missing values, outliers, normality of their distributions and pairwise linearity prior to analysis [40].

We used Pearson's correlations (2-tailed) to test the relationship between the average attractiveness judgements of the White Scottish and Black South African participants (SPSS v21). To do so, we calculated correlated averages (i.e. correlating average attractiveness judgements) and not averages of correlations (i.e. correlating individual attractiveness judgements and then averaging the correlation coefficients; [41], [42]) for two reasons: (a) we were interested in the strength of the correlation between different groups (e.g. African and Scottish observers), not between individuals within the groups; and (b) the groups had similar inter-rater reliabilities and number of raters.

To address the second aim of the study, we compared a limited set of the Pearson's correlation coefficients using Fisher's Z test [43], [44] to compare independent correlation coefficients (e.g. correlation coefficients for different populations) and Steiger's Z test [43] to compare dependent correlation coefficients (e.g. correlations coefficients within a population). Z values were converted to p values (2-tailed; [45]). We compared only a planned set of correlation coefficients and, where necessary, adjusted the alpha level using the Bonferroni correction method (α′ = 0.05/*k*, where α′ is the adapted significance level and *k* is the number of tests), to guard against type one errors associated with multiple testing.

### Results and Discussion

Familiarity with the image participants significantly increased their attractiveness judgements (Material S1). We therefore excluded all individual ratings where observers knew the image participants (2.5% of cases). Inter-rater reliability of attractiveness judgements was high for all groups (Cronbach's α>0.92; Table S1). All attractiveness variables were normally distributed (2-tailed critical z score = ±3.29) with no univariate outliers at p<0.001 (2-tailed critical z score = ±3.29; [40]).

African and Scottish observers' attractiveness judgements were significantly correlated for all faces (r = 0.623, p<0.001, N = 179), but African and Scottish observers agreed more strongly on what was attractive in Scottish faces (r = 0.747, p<0.001, N = 83; male faces only: r = 0.696, p<0.001, N = 40; female faces only: r = 0.791, p<0.001, N = 43) than what was attractive in African faces (r = 0.487, p<0.001, N = 96; male faces only: r = 0.365, p = 0.012, N = 47; female faces only: r = 0.534, p<0.001, N = 49). Fisher's Z test indicate that cross-cultural agreement was significantly higher for Scottish than for African faces (Fisher's Z = 2.87, p = 0.004). African and Scottish observers also showed slightly stronger agreement in what is attractive in female (r = 0.660, p<0.001, N = 92) than in male faces (r = 0.536, p<0.001, N = 87), but not significantly so (Fisher's Z = 1.39, p = 0.16; single sex judgements of opposite sex faces produced similar results: Material S2). All significant correlations were still significant at Bonferroni adjusted α = 0.007 (0.05/7).

These results provide further evidence of the universality of facial attractiveness preferences, but also highlight that the ethnicity of the target face can influence cross-cultural agreement in attractiveness preferences to some extent. In line with our prediction, African and Scottish participants agreed significantly more strongly when judging European facial features (which both groups are very familiar with) than when judging African facial features (which only the African observers are really familiar with). Although African and Scottish participants agreed somewhat more highly on what was attractive in female than in male faces (especially for African faces), overall the gender of the target face did not have a significant influence on cross-cultural agreement in attractiveness preferences.

## Experiment 2

Experiment 1 found that African and Scottish observers agreed significantly more strongly on what is attractive in Scottish compared to African faces. The question now remains: why is there such discordance in the cross-cultural attractiveness judgements of African faces? Do Scottish and African observers rely on different facial cues when judging African faces for attractiveness? Both Europeans and Africans use shape (e.g. [14], [37], [39]) and skin colour (e.g. [37], [38], [46]–[48]) cues when judging the health and attractiveness of their own ethnicity faces. African faces, however, have much higher variation in skin colour compared to European faces. African observers will also be relatively more familiar with the full range of skin colour cues in African faces compared to Scottish observers. One would therefore expect that African observers would rely more heavily on skin colour cues when judging African faces relative to Scottish observers. Scottish observers, on the other hand, are expected to rely more heavily on shape cues. Indeed, Strom et al. [49] found that Black observers' racial prototypicality ratings of Black faces were more responsive to skin colour, while White observers' ratings were more responsive to facial structure. African and Scottish observers might also utilize different shape cues when judging attractiveness, given their cultural differences in visual experience and the utility of the information. The aim of experiment 2 is therefore to determine which shape and skin colour cues contribute to African and Scottish observer's judgements of facial attractiveness in African faces. We will utilise geometric morphometrics —a technique that objectively captures the geometry (and therefore size and shape) of an object by means of morphometrics points or landmarks― and reflectance spectrophotometry to determine objective measures of shape and colour cues respectively. Both techniques have been successfully used in previous studies to assess the association between facial cues and attractiveness (e.g. [37], [50]).

### Methods

#### Measurements

We used the African image set with corresponding attractiveness judgements described in experiment 1.

Participants' facial skin colour was measured on three different points (forehead, left cheek and right cheek) in CIELab colour space: CIELab L*(luminance axis), CIELab a* (green-red axis) and CIELAb b* (blue-yellow axis) using a Konica Minolta CM2600d spectrophotometer.

#### Image and statistical analyses

To determine the face shape components, we manually delineated the African facial images by defining 119 feature points and aligned these images according to interpupillary distance in PsychoMorph [51]. These delineated images were then subjected to sex-specific Principal Component Analyses (PCA) in PsychoMorph [52]. In accordance with previous work [38], [50], we retained all principal components with eigenvalues greater than the average eigenvalue. PCAs were also used to reduce the average CIELab L*, a* and b* measurements to sex-specific colour components (SPSS v21); all principal components with eigenvalue >1 were retained. We fitted separate linear regressions, with attractiveness as the dependent variable and face shape and colour components as the independent variables, to determine which shape and colour cues predict African and Scottish observer's attractiveness judgements (SPSS v21). Significant and marginally significant (p≤0.08) shape and colour components were visualised using PsychoMorph by (a) producing sex-specific composite images, which consists of 10 individual images averaged together (b) averaging the five highest and five lowest images for the particular component to produce a high and low average image (prototype) for that component, and (c) transforming the composite images 100% towards both the high and low average images [53], [54].

### Results and Discussion

All variables were normally distributed (two-tailed critical z score = ±3.29, p = 0.001), except for CIELab a* for female faces (skewness z score = −5.62; kurtosis z score = 9.69; [40]). The removal of one outlier successfully normalised CIELab a* (skewness z = −2.42; kurtosis z = 3.18), leaving 44 cases for the female analysis. None of the other variables had univariate outliers at p = 0.001 (two-tailed critical z score = 63.29; [40]). Eleven principal components were retained from the female shape PCA, which together explained 83.16% of the variance in female face shape; Ten principal components were retained from the male shape PCA, which explained 81.45% of the variance in male face shape. The female skin colour PCA produced one colour component with eigenvalue >1, which explained 73.90% of the variance in skin colour. Higher values for this colour component indicate a lighter (0.92), yellower (0.95) and redder (0.68) skin tone. The male skin colour PCA produced one colour component with eigenvalue >1, which explained 96.03% of the variance in skin colour. Higher values for this colour component indicate a lighter (0.98), yellower (0.99) and redder (0.97) skin tone. All the PCA components were normally distributed (two-tailed critical z score = ±3.29, p = 0.001) and appeared to be linearly related to attractiveness.

We fitted four simultaneous linear regressions (i.e. male and female faces; African and Scottish observers), with attractiveness as the dependent variable and the shape and colour components as independent variables. Collinearity diagnostics identified no multicollinearity in any of the regression analyses (variance inflation factor <1.5). In the first analysis, colour component 1 significantly predicted African observers' attractiveness judgements of African female faces, while shape component 9 marginally predicted these attractiveness judgements (Table 2). In the second analysis, shape components 4, 8 and 9 significantly predicted Scottish observers' attractiveness judgements of African female faces (Table 2). In the third analysis, only colour component 1 significantly predicted African observers' attractiveness judgements of African male faces (Table 2). No shape or colour components significantly predicted Scottish observers' attractiveness judgements of African male faces (Table 2).

**Table 2:** Results from regression analyses for African and Scottish observers' attractiveness judgements of African faces.

	β	F/t	P	Effect size	95% CI	
					Lower	Upper
*African ratings of African female faces*						
Model		1.642	0.131	0.389		
Colour component	0.410	2.530	0.017	0.414	0.023	0.440
Shape component 9	−0.269	−1.812	0.080	−0.309	−0.036	0.008
*Scottish ratings of African female faces*						
Model		2.921	0.008	0.531		
Shape component 9	−0.472	−3.627	0.001	−0.546	−0.056	−0.012
Shape component 8	−0.324	−2.460	0.020	−0.404	−0.054	−0.003
Shape component 4	−0.259	−2.083	0.046	−0.350	−0.024	0.001
*African ratings of African male faces*						
Model		2.374	0.031	0.474		
Colour comp 1	0.472	2.854	0.008	0.473	0.037	0.512
*Scottish ratings of African male faces*						
Model		0.792	0.647	0.231		

Results obtained using simultaneous regression method. F statistics are indicated for the overall model and t statistics for individual coefficients. Effect size: R2 (model); Partial eta squared (variables). β indicates the standardized beta coefficient and CI the confidence interval based on 1000 bootstrap samples. Only significant and marginally significant (p≤0.08) coefficients are indicated. Higher values for the colour components indicate a lighter, yellower and redder complexion. High values for the shape components seem to indicate: higher facial adiposity and/or robustness (shape component 9); lower femininity and/or neoteny (shape component 8 and 4).

The shape and colour components are visualised in Figure 1. Briefly, positive values for the male and female colour component indicate a lighter, yellower and redder complexion than negative values. Negative values for female shape component 9 seem to indicate relatively lower facial adiposity (e.g. lower facial fatness; [39]) and/or robustness than positive values. Negative values for female shape components 8 and 4 seem to indicate a more feminine (e.g. smaller chin, higher cheekbones; [14]) and more neotenous (e.g. large eyes, small nose; [55]) appearance, which surprisingly also had thinner lips. These results indicate that African observers rely more heavily on colour cues when judging attractiveness in own ethnicity faces, preferring a lighter, yellower and redder complexion in both male and female African images. African observers also seemed prefer lower facial adiposity and/or robustness in female faces to some extent. Scottish observers on the other hand, seem to rely more heavily on shape cues when judging female African faces, preferring a lower facial adiposity/robustness and a more feminine, neotenous appearance.

## General Discussion

Consistent with the meta-analysis by Langlois et al. [4], we found significant agreement between African and Scottish observers in their facial attractiveness preferences, given the significant correlations between the mean attractiveness judgements (and the high inter-rater reliability across attractiveness judgements; Table S1) of the different participant groups. The observed correlation between African and Scottish observers' attractiveness judgements (r = 0.62) was similar to previously reported correlations between populations influenced by Western culture, for example Americans and Koreans (r = 0.64; [8]) and Americans, Brazilians and Russians (average r = 0.64; [6]). These results provide further evidence for significant cross-cultural agreement in attractiveness preferences.

Despite significant general agreement in facial attractiveness preferences between African and Scottish observers, we did observe fine-scale differences in their attractiveness preferences. African and Scottish observers agreed significantly more strongly when judging Scottish faces than when judging African faces. This finding is in line with the proposal that observers should show higher cross-cultural agreement if the target faces are familiar to both groups (e.g. Scottish faces), compared to when the target faces are less familiar to both (or one) of the groups (e.g. African faces). There are several plausible reasons why familiarity/perceptual experience with a specific group of faces should increase cross-cultural agreement in attractiveness preferences. First, increased perceptual experience with other-ethnicity faces could reverse the own-race bias, not only for discrimination and recognition ability, but also for other perceptual expertise such as attractiveness judgements. Second, more perceptual experience with a given ethnicity could lead to the development of a more defined ‘prototype’ for that ethnicity. Since both Africans and Scottish observers are very familiar with European facial features, they both most likely have a more defined and therefore closely aligned ‘prototype’ for European faces. Due to their limited exposure to African faces, Scottish observers most likely don't have a clearly defined ‘prototype’ for African faces, which would as a consequence not be very closely aligned to the African's observers' ‘prototype’ for African faces. Third, increased interracial contact could also increase knowledge of the utility of information in a particular group of faces. For example, due to their close contact with White students, African university students likely learn the facial features that convey low attractiveness or ill health in European faces, while the reverse is probably not true in Scottish university students.

To our knowledge no previous study has tested the hypothesis that target face ethnicity influences cross-cultural agreement in attractiveness preferences explicitly, but previous work provides some support for a positive association between cross-cultural agreement in attractiveness judgements and the familiarity of the facial features. Zebrowitz et al. [8] found higher cross-cultural agreement in attractiveness judgments within race (e.g. more familiar) than between race (e.g. less familiar). Similarly, Jones and Hill [6] found higher cross-cultural agreement in attractiveness judgments within the Western student cluster (White US, Brazilian and Russian students) and the Indian adult cluster (Paraguayan and Venezuelan Indians) than between the two clusters. White US, Brazilian and Russian students most likely have more exposure to each other's facial features than to Paraguayan and Venezuelan Indian facial features. Paraguayan and Venezuelan Indian populations do not have contact with each other [6], but most likely share similar facial features given their fairly recent divergence [56] that will indirectly increase the familiarity with the other population's facial features. Zebrowitz et al. [9] also reported higher cross-cultural agreement in attractiveness judgments within race than between race. Moreover, they found higher cross-cultural agreement in attractiveness preferences for American faces (r = 0.50) than Tsimane faces (r = 0.29), although this finding might be attributed to the fact that American faces were selected to represent the extremes of attractiveness while Tsimane faces were not.

We conducted a second experiment to further investigate the discordance between African and Scottish observers' attractiveness judgements of African faces. Results show that African observers rely more heavily on skin colour cues when judging African faces, while Scottish observers rely more heavily on shape cues. These findings are in line with our prediction that African observers would rely more heavily on skin colour cues than Scottish observers, given that skin colour is a more variable and salient cue in African populations and that African observers are more familiar with the full range of skin colour in African faces. African observers also likely have a better understanding of the association between African skin colour and traits such as fertility and health. Previous work on racial prototypicality ratings showed that Black observers are more responsive to skin colour while White observers are more responsive to facial structure [49], providing further support for our findings. African observers preferred a significantly lighter, yellower and redder complexion for both male and female African faces. Scottish observers, on the other hand, showed a strong preference for skinnier/less robust African female faces and a slightly weaker preference for a more feminine/neotenous-looking African female faces. Interestingly, African observers also preferred skinnier/less robust African female faces (albeit only marginally), indicating that facial adiposity/facial robustness plays a crucial role in female attractiveness judgements across cultures. Coetzee et al. [37] also reported a preference for skinnier African women amongst African university students. The preference for skinnier women is inconsistent with traditional African ideals and low resource availability but consistent with modern African media ideals [37], [57]. There were no significant predictors for Scottish observers' judgements of African male attractiveness. We should point out that we did not directly test African and Scottish participants' attractiveness preferences for the specific shape and colour components indicated in Figure 1, which limits the conclusions that can be drawn from these latter results somewhat.

We did not find significant support for the proposed relationship between target face gender and cross-cultural agreement; African and Scottish observers showed higher agreement for female, compared to male target faces, but not significantly so. One might argue that we did not observe a significant difference in cross-cultural agreement for male and female faces because we combined male and female attractiveness judgements instead of using only opposite sex judgements. Single sex judgements of opposite sex faces, however, produced similar results, in that African and Scottish observers did not show significantly higher agreement for female, compared to male target faces (Material S2).

In summary, our results show significant general agreement between the attractiveness judgements of African observers from South Africa and Scottish observers, providing further evidence for strong cross-cultural agreement in facial attractiveness preferences. Nevertheless, we find significantly stronger cross-cultural agreement in attractiveness preferences for Scottish, compared to African, faces. The discordance between Scottish and African observers' attractiveness judgements can be partly explained by their varying reliance on facial shape and colour cues.

## Supporting Information

Table S1 Inter-rater reliability of attractiveness judgements.
(DOCX)

Material S1 Effect of familiarity on attractiveness judgements.
(DOCX)

Material S2 Single sex judgements of opposite sex faces.
(DOCX)
