# Supplementary material for: Cross-Cultural Agreement in Facial Attractiveness Preferences: The Role of Ethnicity and Gender
Source: PLoS One. 2014 Jul 2;9(7):e99629. doi: 10.1371/journal.pone.0099629 (PMC4079334; doi:10.1371/journal.pone.0099629)
Supplement: Table S1 — Inter-rater reliability of attractiveness judgements. (DOCX) [file pone.0099629.s001.docx]

**Cross-cultural agreement in facial attractiveness preferences: the role of ethnicity and gender.**

Coetzee, V., Greeff, J.M. Stephen, I. D. and Perrett, D.I.

**Supporting information**

*Table S1. Inter-rater reliability of African and Scottish participants’ attractiveness judgements*

|  | African | Scottish |  | African & Scottish |
| --- | --- | --- | --- | --- |
| *African images* | | | | |
| Female | 0.93 (30) | 0.97 (32) |  | 0.96 (62) |
| Male | 0.93 (31) | 0.93 (30) |  | 0.94 (61) |
| *Scottish images* | | | | |
| Female | 0.95 (29) | 0.95 (26) |  | 0.97 (55) |
| Male | 0.92 (27) | 0.94 (23) |  | 0.96 (50) |

*Cronbach’s alpha coefficient of reliability for each group and the combination of rater groups. Sample sizes are indicated in brackets.*
