# Supplementary material for: Cross-Cultural Agreement in Facial Attractiveness Preferences: The Role of Ethnicity and Gender
Source: PLoS One. 2014 Jul 2;9(7):e99629. doi: 10.1371/journal.pone.0099629 (PMC4079334; doi:10.1371/journal.pone.0099629)
Supplement: Material S1 — Effect of familiarity on attractiveness judgements. (DOCX) [file pone.0099629.s002.docx]

**Cross-cultural agreement in facial attractiveness preferences: the role of ethnicity and gender.**

Coetzee, V., Greeff, J.M. Stephen, I. D. and Perrett, D.I.

**Supporting information**

*Material S1. Effect of familiarity on attractiveness judgements*

We used a paired sample t-test to test whether image participants were judged more attractive by observers that reported knowing them than by observers that reported not knowing them. The test compared (a) individual attractiveness judgements of observers who knew the image participants, with (b) the average attractiveness of that image participant as judged by observers who did not know them.

There were 251 individual cases where observers reported knowing individual image participants in the pooled data set. Observers that knew image participants judged them significantly more attractive (Mean±SD = 3.30±1.62) than the average attractiveness judgement of observers who did not know them (Mean±SD = 2.57±0.76), paired t test: t250 = 7.98, p < 0.001. We therefore excluded individual ratings where observers knew the image participants (2.48% of cases) from all subsequent analysis.
