# Supplementary material for: Cross-Cultural Agreement in Facial Attractiveness Preferences: The Role of Ethnicity and Gender
Source: PLoS One. 2014 Jul 2;9(7):e99629. doi: 10.1371/journal.pone.0099629 (PMC4079334; doi:10.1371/journal.pone.0099629)
Supplement: Material S2 — Single sex judgements of opposite sex faces. (DOCX) [file pone.0099629.s003.docx]

**Cross-cultural agreement in facial attractiveness preferences: the role of ethnicity and gender.**

Coetzee, V., Greeff, J.M. Stephen, I. D. and Perrett, D.I.

**Supporting information**

Material S2: *Single sex judgements of opposite sex faces*

To test whether the aggregation of male and female attractiveness judgements obscured a significant difference in cross-cultural agreement for male and female faces, we redid the analysis using only single sex judgements of opposite sex faces. As with the combined attractiveness judgements, African and Scottish participants agreed more strongly on what is attractive in female (r=0.574, p≤0.001) than in male faces (r=0.476, p≤0.001), but not significantly so (Fisher’s z=0.89, p=0.37).
